# Rotavirus VP6 Adjuvant Effect on Norovirus GII.4 Virus-Like Particle Uptake and Presentation by Bone Marrow-Derived Dendritic Cells In Vitro and In Vivo

**DOI:** 10.1155/2020/3194704

**Published:** 2020-01-23

**Authors:** Kirsi Tamminen, Suvi Heinimäki, Timo Vesikari, Vesna Blazevic

**Affiliations:** Vaccine Research Center, Faculty of Medicine and Health Technology, Tampere University, Biokatu 10, FI-33520 Tampere, Finland

## Abstract

We have previously shown that rotavirus (RV) inner capsid protein VP6 has an adjuvant effect on norovirus (NoV) virus-like particle- (VLP-) induced immune responses and studied the adjuvant mechanism in immortalized cell lines used as antigen-presenting cells (APCs). Here, we investigated the uptake and presentation of RV VP6 and NoV GII.4 VLPs by primary bone marrow-derived dendritic cells (BMDCs). The adjuvant effect of VP6 on GII.4 VLP presentation and NoV-specific immune response induction by BMDC *in vivo* was also studied. Intracellular staining demonstrated that BMDCs internalized both antigens, but VP6 more efficiently than NoV VLPs. Both antigens were processed and presented to antigen-primed T cells, which responded by robust interferon *γ* secretion. When GII.4 VLPs and VP6 were mixed in the same pulsing reaction, a subpopulation of the cells had uptaken both antigens. Furthermore, VP6 copulsing increased GII.4 VLP uptake by 37% and activated BMDCs to secrete 2-5-fold increased levels of interleukin 6 and tumor necrosis factor *α* compared to VLP pulsing alone. When *in vitro*-pulsed BMDCs were transferred to syngeneic BALB/c mice, VP6 improved NoV-specific antibody responses. The results of this study support the earlier findings of VP6 adjuvant effect *in vitro* and *in vivo*.

## 1. Introduction

Rotavirus (RV) and norovirus (NoV) account for the majority of acute viral gastroenteritis (AGE) cases globally [1]. Introduction of RV vaccination into national immunization programs has reduced the incidence of RV AGE [2], but vaccine against NoV is still under development [3]. Due to the challenges in propagation of NoV in cell culture, excluding live attenuated vaccines [4], NoV vaccine development is largely based on virus-like particles (VLPs) [3], which are spontaneously formed after the expression of NoV major capsid protein VP1 *in vitro*, e.g., in a baculovirus-insect cell expression system [5]. Nonlive RV-subunit vaccines are under development due to the safety concerns and low efficacy of live attenuated vaccines in developing countries [6]. The most abundant and conserved RV protein, VP6, forms the intermediate layer of triple-layered RV particle [7] and although it does not induce classical neutralizing antibodies, intracellular neutralization by VP6-specific polymeric immunoglobulin (Ig)A [8] and VP6-specific CD4+ T cells are associated with protection in mice [9]. Highly immunogenic VP6 proteins can form various nanostructures *in vitro* [10], and VP6 has been suggested as the next-generation nonlive vaccine candidate against RV [11–13]. Our group has combined oligomeric VP6 nanostructures with NoV VLPs to generate nonlive subunit combination vaccine against NoV and RV [14, 15].

Preclinical and clinical studies have shown that immunization with NoV VLPs leads to a robust antibody response with surrogate neutralization capacity—a factor that correlates with protection [16–18]. However, the heterogenicity of numerous NoV genotypes [19] and the antigenic evolution of the most prevalent NoV genotype, GII.4 [20], make vaccine development challenging [3]. Adjuvant is an option to strengthen and broaden NoV immune responses, and NoV vaccine candidate adjuvanted with aluminum hydroxide and monophosphoryl lipid A has been tested in phase IIb clinical trials [21]. However, due to the local and systemic adverse events associated with adjuvanted vaccines [22], our hypothesis is that in the pediatric NoV vaccine candidate we developed, extremely immunogenic VP6 protein may serve to substitute the external adjuvant. To this end, we have shown that RV VP6 in our combination vaccine has an adjuvant effect on NoV immune responses *in vitro* and *in vivo* [14, 23–25]. The adjuvant mechanism has been studied in immortalized cell lines used as antigen-presenting cells (APCs), namely, RAW macrophages and JAWSII dendritic cells (DCs), and the results suggested that VP6 acts as an immunomodulator and immunostimulator and facilitates NoV VLPs internalization by the APCs [23].

DCs are professional APCs that play a principal role in both T and B cell immune responses leading to adaptive immunity [26]. DCs capture the antigens and, after processing them, present the digested proteins as short peptides within MHC class I and II molecules to effector T cells [27]. DCs modulate the cytokine environment by exerting cytokines and chemokines, which attract other cells to the inflammation site, and naïve T and B cells within lymph nodes [28]. Mouse bone marrow-derived dendritic cells (BMDCs) have been used as a research tool in studies investigating antigen uptake and presentation *in vitro* [29–32], and VLPs derived from different viruses have been shown to be uptaken and processed by the BMDCs [30, 32, 33]. Furthermore, antigen-pulsed BMDCs can be used *in vivo* to examine the role of DCs in the generation of immunity against various infectious diseases [29, 34, 35] as well as immunotherapeutic agents [36, 37]. In the present study, we used BALB/c mouse primary BMDCs to investigate the ability of these cells to uptake, process, and present NoV and RV antigens *in vitro* and to generate an immune response *in vivo*. Furthermore, the adjuvant effect of RV VP6 in enhancing NoV VLP uptake and immune response induction by the pulsed BMDCs was investigated.

## 2. Materials and Methods

### 2.1. Antigens

NoV GII.4 VLPs (accession no. AF080551) and RV VP6 proteins (accession no. GQ477131) were produced in a baculovirus-insect cell expression system and purified through sucrose-gradients as previously described [14, 38]. Final purification was conducted by consecutive ultrafiltrations containing dissociation and reconstitution steps as described earlier for RV VP6 [39]. Briefly, the structure of VP6 nanotubules was dissociated in 80 mM sodium acetate (pH 3.0, overnight at +4) and filtrated using Vivaspin 300K centrifugal filter devices (Sartorius AG, Frankfurt, Germany) to remove large impurities. The filtrate was then subjected to another ultrafiltration with 30K filter unit (Amicon Ultra-15, Millipore Corporation, Carrigtwohill, Ireland), and the retentate containing VP6 was collected. The tubular form of VP6 proteins was restored by exchanging buffer to sterile phosphate buffered saline (PBS, Lonza, Verviers, Belgium), pH 7.3-7.5. Finally, the preparation was concentrated with 30K filter units (Millipore). Similar purification step was conducted for NoV VLPs. VLPs were dissociated in 50 mM ammonium acetate (pH 9.0) at low protein concentration (0.1 mg/ml) [40]. After overnight incubation, the preparation was ultrafiltered (3000×g) using Vivaspin 300K filter units (Sartorius). The filtrate was then subjected to another filtration with Millipore 30K filter units (4000×g) leaving GII.4 dimers in retentate. Finally, VLP structures were reconstructed with sterile PBS (pH 7.3-7.5, Lonza), and the final product was concentrated using 50K filter units (Millipore).

**Figure 1:**
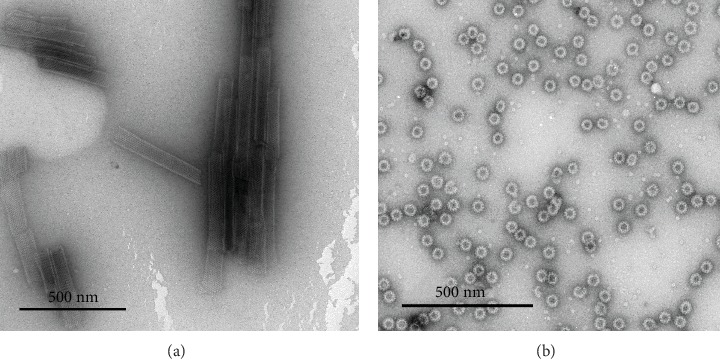
Structure and integrity of the purified RV VP6 and NoV GII.4 VLPs. The proteins were produced in a baculovirus-insect cell system and purified by sucrose gradients followed by ultrafiltrations of dissociated proteins as described in Materials and Methods. Electron microscopy images of reconstructed rotavirus VP6 nanotubes (a) and norovirus GII.4 virus-like particles (b) examined by a FEI Tecnai F12 electron microscope (Philips Electron Optics) at a magnification of 11 000x and 12 000x, respectively, after negative staining with 3% uranyl acetate, pH 4.6. Scale 500 nm.

The final products were characterized as described earlier [39, 41]. Accordingly, the protein concentration was measured using a BCA protein assay kit (Pierce), and morphology of purified RV VP6 nanotubes and NoV VLPs were observed using electron microscopy (EM) (Figure 1). In addition, saliva-based histoblood group antigen (HBGA) binding assay [42] was conducted to confirm intact NoV VLPs HBGA binding (data not shown). Another batch of NoV GII.4 VLPs (accession no. BAG70446), used as antigen in splenocyte coculture assay (described below), were produced as previously described [43].

### 2.2. Isolation and Generation of BMDCs

Primary bone marrow (BM) cells were isolated and cultured according to the published procedures [44, 45]. Femurs and tibiae of naïve BALB/c OlaHsd (Envigo RMS BV, Horst, the Netherlands) mice were cut with scalpel from each end and flushed with cold PBS to collect BMs, which were passed through 70 *μ*m cell strainers (Becton-Dickinson, BD, Franklin Lakes, NJ, USA). The single cell cultures were suspended in complete medium (CM, RPMI-1640 supplemented with 100 U/ml penicillin, 100 *μ*g/ml streptomycin, 50 *μ*m 2-mercaptoethanol, 2 mm l-glutamine, and 10% fetal bovine serum (FBS), all from Sigma-Aldrich) and subjected to centrifugation for 10 min, 300×g. The cells were seeded on nontreated 14.2 mm sterile petri dishes (VWR, Radnor, PA, US) at 1 × 10^6^ cells/ml (10 ml/plate) in CM/10% FBS supplemented with a recombinant mouse granulocyte-macrophage colony-stimulating factor (GM-CSF, Abcam, Cambridge, UK) at 20 ng/ml and cultured for 8 days (37°C and 5% CO_2_). Five milliliters of fresh medium containing GM-CSF (20 ng/ml) was added on days 4 and 7, and nonattached cells were harvested from plates with gentle washing on day 8. The cells were tested for the expression of CD11c molecules, costimulation molecules (CD80, CD86), and MHC class II (I-A/I-E) by surface staining and flow cytometry as described below.

### 2.3. BMDC Pulsing

Frozen BMDCs were thawed, washed two times with 15 ml of CM/10% FBS, and seeded at 2‐2.5 × 10^6^ cells/ml in 24-well nontreated cell culture plates (Corning Costar) for pulsing. RV VP6 and GII.4 VLPs were added to the cell cultures as single or mixed antigens at the concentration of 100 *μ*g/ml. Lipopolysaccharide (LPS) (Sigma-Aldrich) was used at a concentration of 1 *μ*g/ml as a positive stimulant for cytokine release assays. Unpulsed cells receiving no antigen were used as negative control. All the pulsing reactions were carried out at +37°C, 5% CO_2_ for 20–22 h. Supernatants of the BMDC cultures were collected and stored at -80°C until use. BMDCs were harvested from the wells and washed 2–3 times with 15 ml CM/10% FBS (300×g, 10 min) to remove the free antigen.

### 2.4. Cell Staining and Flow Cytometry

For the staining of cell surface and internalized molecules, previously published procedures with some modifications were utilized [23]. Briefly, BMDCs were washed with staining buffer (Becton Dickinson, BD, San Jose, CA) following a blocking step on ice for 10 min using rat anti-mouse CD16/CD32 (Fc Block, Clone 2.4G2, BD). After washing with DPBS (Lonza), dead cells were stained with Horizon™ Fixable Viability Stain 780 (FVS780, BD) for 10 min at RT in the dark, washed, and were divided into staining reactions (0.25–0.5 × 10^6^ cells/reaction). For the analysis of cell surface molecule expression, the following anti-mouse antibodies were used for staining: anti-CD11c (HL3) conjugated to phycoerythrin (PE), CD80 (16-10A1) conjugated to peridinin chlorophyll-cyanine5.5 (PerCP-Cy5.5), CD86 (GL1) conjugated to PE-Cy7, and I-A/I-E (M5/114.15.2) conjugated to Alexa Fluor 647 (all purchased from BD). The antibodies were added to cell suspensions and incubated on ice for 30 min. For intracellular staining, the cells were subjected to fixation and permeabilization using a Cytofix/Cytoperm Plus kit (BD) according to the manufacturer's instructions. VP6 proteins were stained with rabbit polyclonal rotavirus group A antibody (GenWay Biotech Inc., San Diego, CA) reacting with FITC-conjugated goat anti-rabbit Ig (BD). For the detection of intracellular GII.4 VLPs, the cells were stained with NoV-positive human serum (diluted 1 : 2000) following PE-conjugated polyclonal anti-human IgG (eBioscience, Thermo Fisher Scientific, Waltham, MA) staining. All the intracellular staining steps were conducted on ice for 30 min. Finally, both surface and intracellularly stained cells were washed twice to remove unbound antibodies and acquired with a FACSCanto II fluorescence-activated flow cytometer (BD).

Surface molecule expression, as well as VP6 and GII.4 VLP internalization, was examined by overlaying histograms of unpulsed and pulsed cells and comparing median fluorescence intensity (MFI) of each population. In addition, dot plots of BMDCs pulsed with GII.4 VLPs alone and in combination with VP6 were created to investigate cell populations positive for internalized proteins. Negative populations were defined using unpulsed BMDCs stained with the same procedures as the GII.4- or VP6-pulsed cells. The data analysis was conducted with FlowJo analysis software (v. 10, Three Star Inc., San Carlos, CA).

### 2.5. Mouse Immunizations

**Figure 2:**
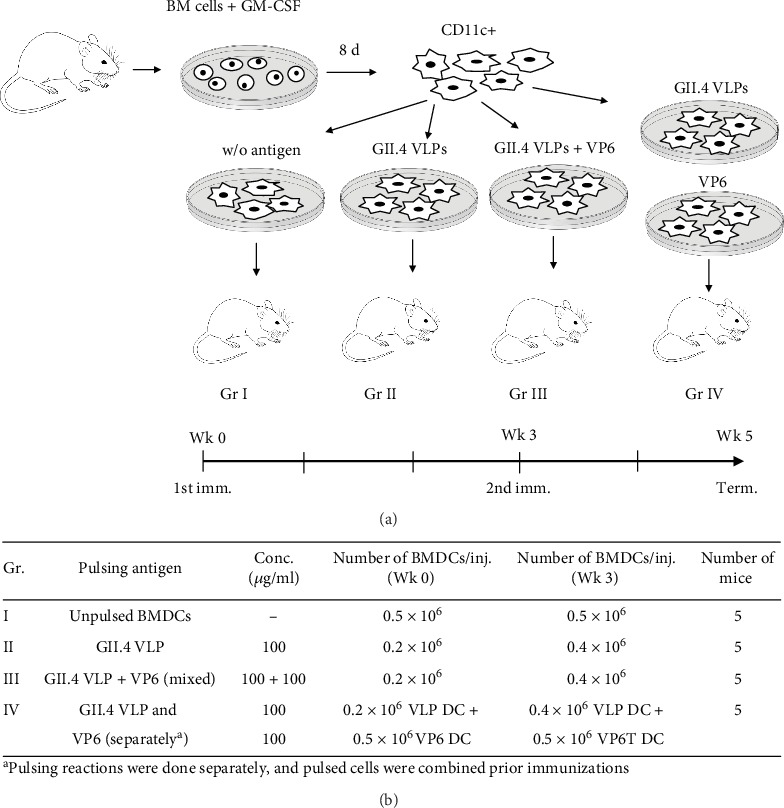
Bone marrow-derived dendritic cell (BMDC) pulsing and experimental immunization groups. (a) Schematic representation of BMDC pulsing with norovirus GII.4 virus-like particles (VLPs) and rotavirus VP6 alone or as mixed antigens. The horizontal arrow illustrates the immunization schedule. (b) Immunization groups, protein concentrations used in pulsing reactions, the number of BMDCs used for immunizations, and the number of mice per immunization group are shown. w/o: without.

Unpulsed BMDCs (Gr I), BMDCs pulsed with GII.4 VLPs (Gr II) or with VLPs and VP6 as a mixture (Gr III) or separately (Gr IV), were administered intramuscularly to 7-week-old female BALB/c OlaHsd mice on study week 0 (Figure 2). Each experimental group contained five mice. The mice received second immunization at study week 3. The mice were euthanized at study week 5, and the sera were collected as described before [46]. In separate experiments, GII.4 VLPs and VP6 were used as protein antigens (10–30 *μ*g/dose) to immunize mice according to our standard protocol [14, 15] to obtain antigen-primed splenocytes for *in vitro* enzyme-linked immunospot (ELISpot) and splenocyte coculture assays (described below). All procedures were authorized and conducted under the guidelines of the Finnish National Animal Experiment Board (permission number ESAVI/10800/04.10.07/2016).

### 2.6. ELISpot Assay

An ELISpot assay was used to enumerate interferon gamma- (IFN-*γ*-) producing T cells of antigen-primed splenocytes in response to stimulation with unpulsed or pulsed BMDCs [45]. Ninety-six-well MultiScreen HTS-IP filter plates (Millipore, Billerica, MA, USA) were coated with anti-mouse interferon IFN-*γ* (Mabtech Ab, Nacka Strand, Sweden) and blocked with CM containing 10% FBS. Unpulsed or pulsed BMDCs were added in CM/10% FBS on plates at 5000, 20 000, and 40 000 BMDCs/well. GII.4 VLPs and VP6 nanotubes were also added as free protein antigens (30 *μ*g/ml), and Concanavalin A (ConA, Sigma-Aldrich) at the concentration of 10 *μ*g/ml was used as a positive control. Splenocytes of GII.4 VLP and VP6 immunized mice were thawed, washed twice, and added (0.2 × 10^6^ cells/well) into the wells followed by ~20 h incubation at +37°C and 5% CO_2_. After discarding the cells, biotinylated anti-mouse IFN-monoclonal antibody (0.5 *μ*g/ml) was added and the spots were developed with alkaline-phosphatase (ALP) conjugated streptavidin (1 : 1000) reacting with BCIP/NBT substrate for 12 min (all reagents from Mabtech). The spots were counted by an ImmunoSpot® automatic CTL analyzer (CTL-Europe GmbH, Bonn, Germany), and the results are expressed as mean spot-forming cells (SFCs) per 10^6^ viable splenocytes of replicate wells.

### 2.7. Splenocytes and BMDC Cocultures

Unpulsed and pulsed BMDCs were cocultured with GII.4 immunized and naïve mouse splenocytes for the detection of antigen-specific antibody production *in vitro.* Splenocytes were thawed, washed, and seeded at 2 × 10^6^ cells/ml (1 ml/well) in 24-well plates (Corning Inc.). GII.4 VLP-pulsed and GII.4 VLP-unpulsed BMDCs were washed three times to efficiently remove free antigen from the cultures and mixed with splenocytes (0.1 × 10^6^ BMDCs/reaction). GII.4 VLPs were added a concentration of 0.05 *μ*g/ml as a control (representing the theoretical concentration of residual free protein after washing). Splenocytes lacking any BMDC or VLP stimulation were also used as negative control. Cells were cultured on a 24-well microplate (Corning Costar) at 37°C and 5% CO_2_ for seven days. Supernatants were collected from the cultures on day 1 and day 7 and tested in enzyme-linked immunosorbent assay (ELISA) as described below to detect GII.4-specific IgG.

### 2.8. IgG, IgG1, and IgG2a ELISA

NoV GII.4 and RV VP6-specific IgG, IgG1, and IgG2a were measured by ELISA as previously described in details [46]. Briefly, GII.4 VLPs and VP6 were used to coat half-area 96-well polystyrene microplates (Corning Inc.) overnight at +4°C. After blocking with 5% milk in PBS, the serum samples (diluted 1 : 100) from each mice or undiluted coculture supernatants were added on plates in duplicates. Secondary antibodies specific for mouse IgG (1 : 4000, Sigma-Aldrich), IgG1 (1 : 6000, Invitrogen, Carlsbad, CA), or IgG2a (1 : 6000, Invitrogen) reacting with OPD substrate (Sigma-Aldrich) were used to detect GII.4- and VP6-specific antibodies from the samples. Victor^2^ microplate reader (Wallack, Perkin Elmer) was used to measure the optical density (OD) values at 490 nm from the plates, and the results were analyzed after subtracting background OD values (blank wells) from each OD reading. Sample giving OD above the cut-off value (mean OD of negative control mice + 3 × SD and at least 0.1 OD) was considered as positive. The results are expressed as the mean of all OD values (±SEM) in the immunization group.

### 2.9. Cytokine ELISA

Quantities of interleukin 6 (IL-6) and tumor necrosis factor *α* (TNF-*α*) in supernatants of BMDC cultures were examined using commercial enzyme-linked immunosorbent assay (ELISA) kits: mouse IL-6 DuoSet (R&D Systems, Minneapolis, MN) and TNF-*α* DuoSet (R&D System) according to the manufacturer's instructions. The supernatants were diluted 1 : 2 and ran as duplicates. The ODs were measured at 490 nm as described above. Standard curves were plotted and used to calculate the cytokine concentration (pg/ml) in the supernatants.

### 2.10. Blocking Assay

In order to determine the surrogate neutralization ability of NoV GII.4-specific antibodies, a blocking assay was conducted according to a published protocol [24] using human saliva type A as the source of HBGAs [42]. Individual mouse sera (diluted 1 : 50) or groupwise pooled sera (titrated from 1 : 50) were preincubated with 0.1 *μ*g/ml GII.4 VLPs for 1 h at +37°C in low-binding tubes (Eppendorf, Hamburg, Germany) prior to adding the preparations on saliva-coated plates. The bound VLPs were detected using human NoV-positive serum (1 : 4000) and horseradish peroxidase- (HPR-) conjugated anti-human IgG antibody (1 : 6000, Novex, Invitrogen) reacting with OPD substrate (Sigma-Aldrich). The OD readings were measured as described above. Wells incubated with VLPs lacking mouse sera were added to each assay to determine the maximum binding OD. The blocking index (%) was calculated as 100% − [(OD wells with VLP‐serum mix/maximum binding OD) × 100%]. The results are expressed as the mean blocking indexes of individual mice for each immunization group or as mean blocking indexes of replicate wells if groupwise pooled sera were used.

### 2.11. Statistical Analyses

The Mann-Whitney test was employed to assess the statistical differences in the observations between immunization groups. All analyses were conducted by IBM SPSS Statistics for Windows (IBM Corp., Armonk, NY), Version 23.0. A statistically significant difference was defined as a *p* value < 0.05.

## 3. Results

### 3.1. Characterization of the BMDCs

**Figure 3:**
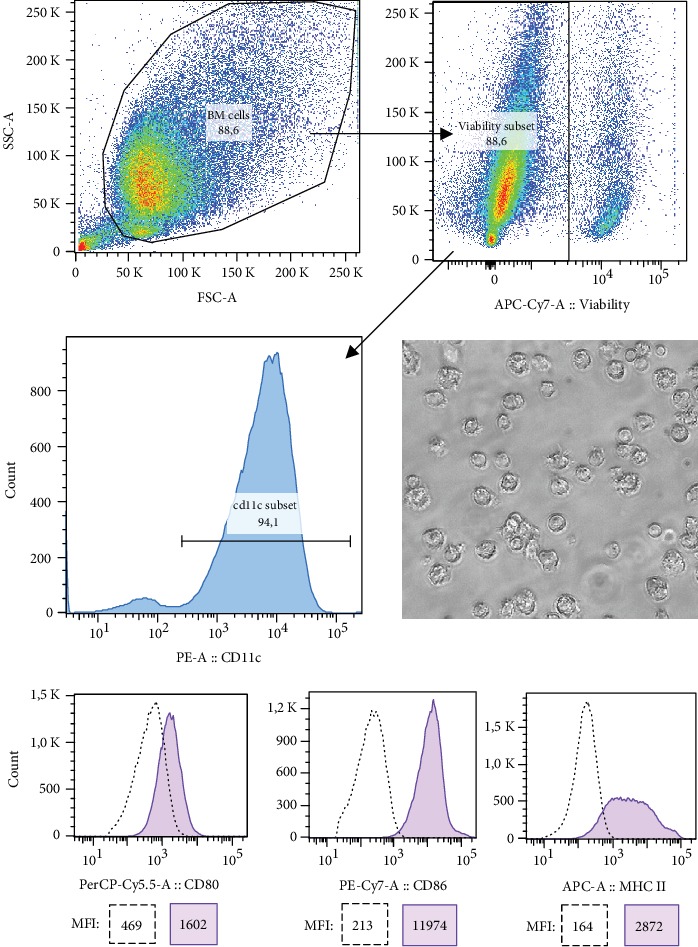
Characterization of granulocyte-macrophage colony-stimulating factor (GM-CSF) generated bone marrow-derived dendritic cells (BMDCs) by flow cytometry analysis. The BMDCs were gated first to exclude debris (left upper panel) and then to exclude nonviable cells (right upper panel). The expression of CD11c is illustrated by a histogram gated on a CD11c-positive subset (left middle panel). Moticam 10000 camera, attached to an inversion light microscope (20x magnification), was used to take an image of generated BMDCs (right middle panel). The expression of CD80, CD86, and MHC class II (I-A/I-E) molecules on surface-stained CD11c+ BMDCs (filled histogram) is illustrated with unstained cells (dotted line) by overlaid histograms (bottom panel) with median fluorescence intensity (MFI) values depicted in the boxes. Results are representative of three independent experiments.

The surface staining confirmed that over 90% cells had CD11c+ phenotype indicating successful generation of BMDCs (Figure 3, left middle panel) [44]. In addition, the cells were observed under an inversion light microscope (Nikon, Minato, Japan) with a Moticam 1000 camera (Motic Microscopy, Wetzlar, Germany) attached, showing typical morphology of BMDCs (Figure 3, right middle panel) [47]. High expression of maturation markers (Figure 3, bottom panel) indicated spontaneous maturation during cultivation with GM-CSF. The BMDCs were frozen in CM containing 10% dimethyl sulfoxide (DMSO) until further use [48]. The viability percent of the freeze-thawed BMDCs was recurrently 80–90% indicating a good recovery of the cells.

### 3.2. BMDCs Internalize GII.4 VLPs and VP6 Nanotubules *In Vitro*

**Figure 4:**
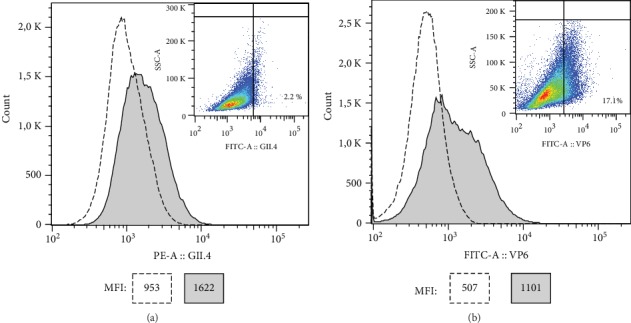
Internalization of norovirus GII.4 virus-like particles (VLPs) and rotavirus VP6 nanotubes. Flow cytometry analysis of norovirus GII.4 VLPs (a) or VP6 nanotubes (b) uptaken by bone marrow-derived dendritic cells (BMDCs) after 22 h incubation with 100 *μ*g/ml of proteins. The intracellular staining of unpulsed BMDCs (dotted line) or BMDC pulsed with the proteins (filled histogram) is shown by overlaid histograms with median fluorescence intensity (MFI) values depicted in the boxes. The percentual uptake of GII.4 VLPs and VP6 nanotubes is shown in the SSC-A versus GII.4-PE (a) or VP6-FITC (b) density plots of BMDCs gated on viable cells. The gates in the density plots are drawn according to the unpulsed BMDCs stained with similar procedures as the pulsed cells (data not shown).

The uptake of GII.4 VLPs and VP6 by BMDCs was examined by flow cytometry after intracellular staining of internalized proteins.  Figure 4 illustrates the shift in the histogram MFI for GII.4 VLPs (Figure 4(a)) and VP6 (Figure 4(b)) after incubation with BMDCs in comparison to untreated BMDCs. The antigen-specific intracellular staining increased for GII.4 VLPs (MFI 1622) and for VP6 (MFI 1101) when compared to untreated, similarly stained, cells (MFI 953 and MFI 507, respectively) indicating that both proteins were internalized by the BMDCs. VP6 nanotubes were more efficiently uptaken than the VLPs as the antigen-positive populations were 2.2% for GII.4 VLP (Figure 4(a), density plot) and 17.1% for VP6 (Figure 4(b), density plot).

### 3.3. GII.4 VLP- and VP6-Pulsed BMDCs Present Antigen and Stimulate T and B Cells *In Vitro*

**Figure 5:**
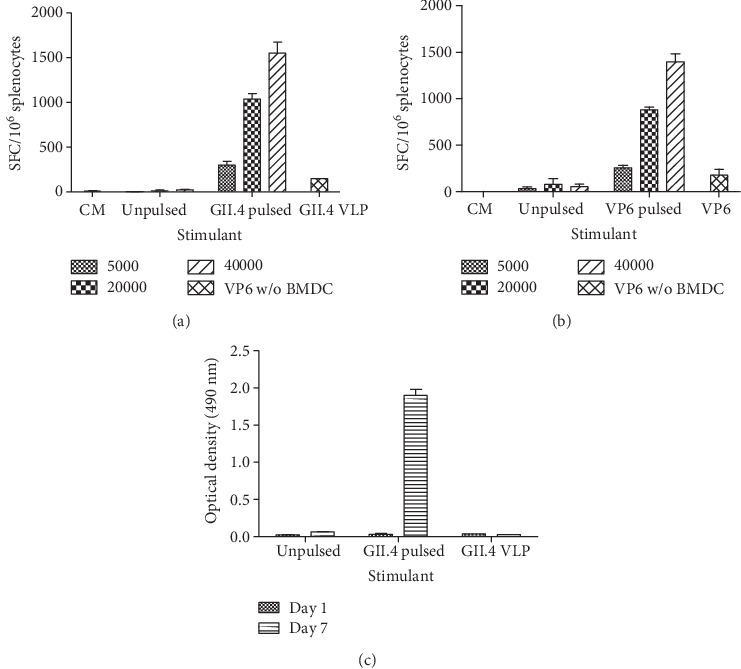
Functionality of norovirus GII.4 virus-like particles (VLPs) and rotavirus VP6-pulsed BMDCs *in vitro*. Splenocytes of mice immunized with GII.4 VLPs (a) or VP6 (b) were analyzed for interferon gamma (IFN-*γ*) production by an ELISpot assay after overnight stimulation with an increasing number of unpulsed, or GII.4 VLP- or VP6-pulsed (100 *μ*g/ml) BMDCs, or native protein antigens (30 *μ*g/ml). Mean IFN-*γ* spot-forming cells (SFCs) per 10^6^ viable splenocytes with standard errors of two to three independent experiments are shown. Unpulsed and GII.4 VLP-pulsed BMDCs were cocultured with GII.4 VLP-immunized mouse splenocytes on a 24-well microplate for seven days, and the supernatant was collected for the measurement of GII.4-specific IgG (c) by ELISA as described in Materials and Methods. GII.4 VLP was added at the concentration of 0.05 *μ*g/ml as a control, representing the theoretical free protein concentration in the BMDC preparations. Shown are the mean optical density (OD) values of two GII.4 VLP-primed mice with standard deviations.

To confirm that GII.4 VLP- and VP6-pulsed BMDC function as APCs, we used unpulsed or pulsed BMDCs as APC in ELISpot IFN-*γ* assay. Robust IFN-*γ* production from antigen-primed splenocytes was detected against GII.4 VLP- (Figure 5(a)) or VP6- (Figure 5(b)) pulsed BMDCs. The magnitude of IFN-*γ* releasing cells increased in relation to the higher number of pulsed BMDCs for both GII.4- (from 298 ± 41 to 1550 ± 125 SFC/10^6^ cells, Figure 5(a)) and VP6- (from 255 ± 25 to 1395 ± 85 SFC/10^6^ cells, Figure 5(b)) specific assay. Unpulsed BMDCs did not activate IFN-*γ* production. GII.4 VLPs or VP6, used as free antigens (30 *μ*g/ml), stimulated considerably lower number of IFN-*γ*-producing cells (145 SFC/10^6^ cells for GII.4 and 175 ± 65 SFC/10^6^ cells for VP6) than pulsed BMDCs, suggesting that GII.4 VLP- and VP6-pulsed BMDCs serve as excellent APCs *in vitro*.

We next determined whether GII.4 VLP-pulsed BMDCs can stimulate B cells for IgG production *in vitro* using GII.4-primed and naïve control mouse splenocytes as responder cells (Figure 5(c)). The coculture supernatants were tested for the presence of GII.4-specific IgG. As a result, no GII.4-specific antibody was present after 1 day of coculture, but after 7 days, a relatively high level of IgG (mean OD 1.899 ± 0.081) was detected in the supernatant of GII.4 VLP-primed mouse splenocytes cocultured with VLP-pulsed BMDCs. In contrast, GII.4 VLP-pulsed BMDCs could not activate naïve mouse splenocytes for antibody production (OD 0.066 ± 0.001, data not shown). To rule out that antigen-specific antibody production was due to the free VLPs in the BMDC preparation, a theoretical residual concentration (0.05 *μ*g/ml of protein) was added as a control stimulant, which failed to induce GII.4-specific IgG in the supernatant (Figure 5(c)). Splenocytes cultivated without BMDCs or VLP stimulation did not produce GII.4-specific antibody (data not shown).

### 3.4. VP6 Improves the Uptake of GII.4 VLPs and BMDC Cytokine Release *In Vitro*

**Figure 6:**
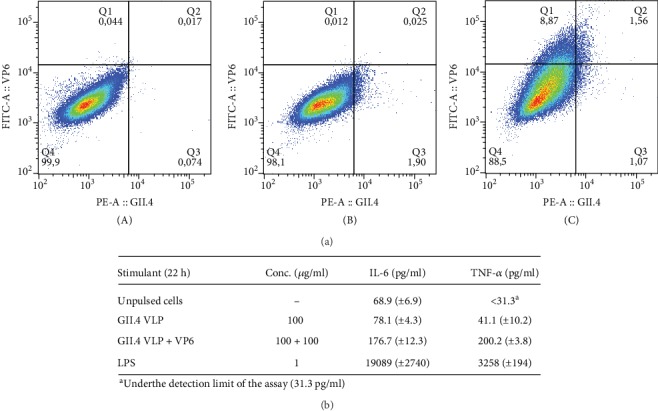
Adjuvant effect of VP6 on GII.4 virus-like particle (VLP) internalization and cytokine release *in vitro.* BMDCs were pulsed ~22 h with 100 *μ*g/ml of GII.4 VLPs alone or in combination with VP6 (100 *μ*g/ml) and intracellularly double-stained with antibodies specific for GII.4 VLPs and VP6. The flow cytometry analysis of unpulsed cells (a, A), GII.4 VLP-pulsed cells (a, B), and GII.4+VP6-pulsed cells (a, C) is represented as dot plots with GII.4-specific fluorescence (PE) plotted on *x*-axis and VP6-specific fluorescence (FITC) plotted on *y*-axis. The level of tumor-necrosis factor *α* (TNF-*α*) and interleukin 6 (IL-6) in BMDC supernatants was measured by ELISA after ~22 h incubation with 100 *μ*g/ml of GII.4 VLPs alone or in combination with VP6 (100 + 100 *μ*g/ml) (b). Lipopolysaccharide (LPS) was used as a positive control. Shown are the mean concentrations (pg/ml) with standard deviations of replicate wells.

The effect of VP6 on GII.4 VLP uptake by BMDC and cytokine release was investigated from cultures of solely GII.4 VLP-pulsed BMDCs in comparison to GII.4 VLP and VP6-copulsed BMDC cultures (Figure 6). Unpulsed and intracellularly stained BMDCs were used to gate the GII.4 and VP6 negative population (Figure 6(a), A). After pulsing with GII.4 VLPs, a small population of GII.4-positive cells (1.9%) was detected (Figure 6(a), B). When VP6 was coadministered with GII.4 VLPs, a 37% increase in the GII.4-positive cells was detected (2.63% of all gated BMDCs) (Figure 6(a), C). Much larger population of copulsed BMDCs had internalized VP6, as 10.4% of BMDCs were positive to intracellularly stained VP6 (Figure 6(a), C). The double staining revealed that 1.56% of the cells were positive for both GII.4 VLP and VP6, indicating that these cells had uptaken both antigens. An increase in inflammatory cytokines, IL-6 and TNF-*α*, was detected in the supernatant of copulsed BMDCs in comparison to GII.4 VLP-pulsed cells (Figure 6(b)). Solely GII.4 VLP-pulsed BMDCs released cytokines closed to the baseline (unpulsed cells), but when VP6 was coadministered with the VLPs, a twofold (from 78.1 to 176.7 pg/ml) and a fivefold (from 41.1 to 200.2 pg/ml) increase in the IL-6 and TNF-*α* levels, respectively, was detected. LPS, used as a positive control, induced robust levels of both cytokines (Figure 6(b)).

### 3.5. GII.4 VLP- and VP6-Pulsed BMDCs Induce Antigen-Specific Immune Responses *In Vivo*

**Figure 7:**
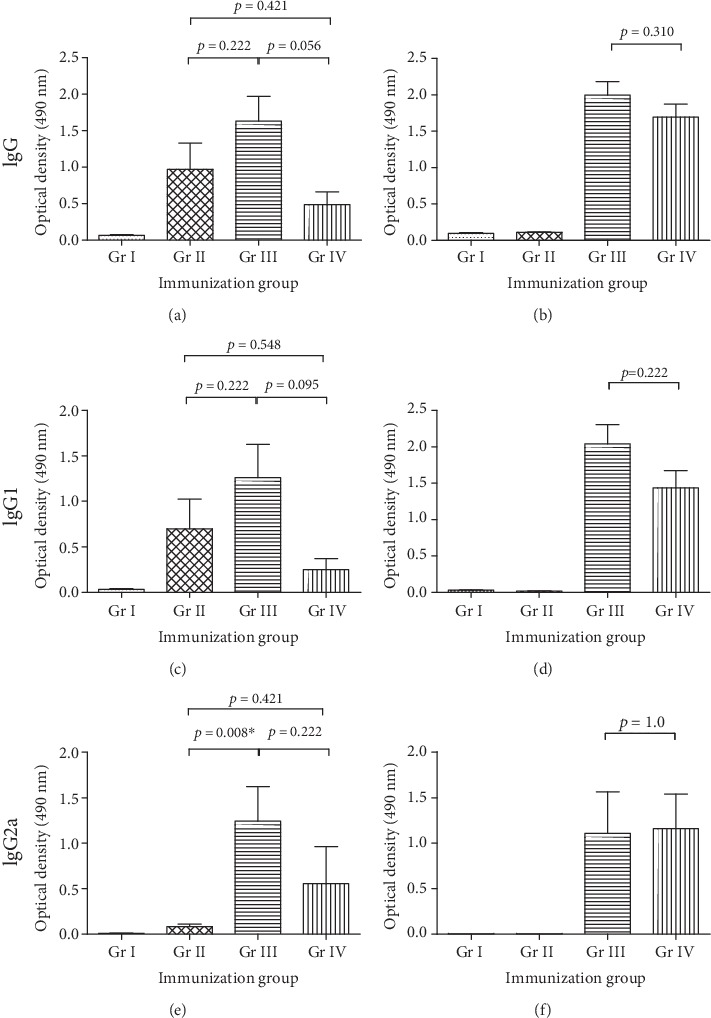
The humoral immune responses generated by *in vitro*-pulsed BMDCs. Norovirus GII.4 virus-like particle- (VLP-) pulsed bone marrow-derived dendritic cells (BMDCs) were transferred intramuscularly two times to mice either alone (Gr II) or in combination with simultaneously (Gr III) or separately (Gr IV) VP6-pulsed BMDCs as described in Materials and Methods. Unpulsed BMDCs were used as negative control cells (Gr I). Mouse termination sera were tested individually (1 : 100 dilution) in GII.4- (left panel) and VP6- (right panel) specific IgG (a, b), IgG1 (c, d), and IgG2a (e, f) ELISAs. Group mean optical density (OD) values at 490 nm with standard error of the means (error bars) are shown. ^∗^Statistically significant *p* value.

Next, we evaluated the ability of GII.4 VLP- and VP6-pulsed BMDCs to induce an immune response in mice. Unpulsed BMDCs, BMDCs pulsed with GII.4 VLPs as single antigen, or coadministered with simultaneously or separately VP6-pulsed BMDCs were transferred to syngeneic BALB/c mice two times (Figure 2), and the antibody responses were evaluated (Figure 7). All immunizations with pulsed BMDCs induced antigen-specific IgG responses in comparison to unpulsed cells. The highest magnitude of GII.4-specific IgG response was detected in the immunization group that received simultaneously VLP- and VP6-pulsed BMDCs (Gr III), although the difference compared to other immunization groups was not statistically significant (*p* = 0.056–0.222, Figure 7(a)). VP6-specific IgG response was similar in groups receiving VP6-pulsed BMDC (*p* = 0.310, Figure 7(b)). Group receiving only GII.4-pulsed BMDCs did not produce VP6-specific IgG confirming the antigen specificity of the assay.

Evaluation of serum levels of IgG subtypes, IgG1 and IgG2a (indicative of Th2 and Th1 responses, respectively), revealed differences between immunization groups. The magnitude of IgG1 followed the same pattern as the total IgG levels; simultaneously GII.4 VLP- and VP6-pulsed BMDCs (Gr III) induced IgG1 responses that trended the highest of all groups (Figures 7(c) and 7(d)). Mice that were immunized with GII.4 VLP-pulsed BMDCs (Gr II) totally lacked IgG2a response, indicating a strong Th2-type response (Figure 7(e)). When VP6 was mixed in the pulsing reaction with the VLPs (Gr III), IgG2a production increased significantly (*p* < 0.008, Figure 7(e)). Separately VP6-pulsed BMDCs improved GII.4-specific IgG2a response to some extent (Gr IV), but not as efficiently as VP6 mixed in the pulsing reaction with VLPs (Gr III) (Figure 7(e)). No difference in the VP6-specific IgG2a responses was detected between the groups receiving simultaneously or separately pulsed BMDCs (Figure 7(f), *p* = 1.0).

### 3.6. BMDCs Pulsed Simultaneously with GII.4 VLP and VP6 Induce GII.4-Specific Blocking Responses

**Figure 8:**
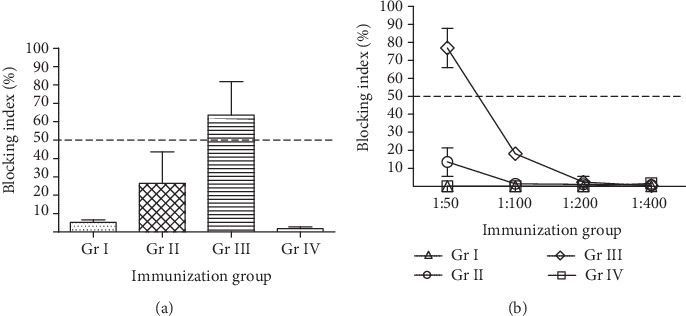
Blocking antibody responses generated by *ex vivo*-pulsed BMDCs. Norovirus GII.4 virus-like particle- (VLP-) pulsed bone marrow-derived dendritic cells (BMDCs) were transferred intramuscularly two times to mice either alone (Gr II) and in combination with simultaneously (Gr III) or separately (Gr IV) VP6-pulsed BMDCs as described in Materials and Methods. The immune sera of mice were tested individually 1 : 50 (a) or as a pool with serial dilutions (b) against GII.4 VLPs in blocking assays utilizing human saliva type A as the source of histoblood group antigens. The blocking index (%) was calculated as follows: 100% − [(OD wells with VLP‐serum mix/maximum binding OD) × 100%]. The error bars represent the standard error between individual mice (a) or two independent assays (b), and the horizontal dashed line illustrates the blocking titer 50%.

Sera from mice immunized with pulsed or unpulsed BMDCs were further tested for blocking activity to determine the surrogate neutralization ability of GII.4-specific antibodies (Figure 8). When individual mouse serum blocking activities were tested, only a group that received simultaneously GII.4 VLP- and VP6-pulsed BMDCs (Gr III) resulted in the mean blocking index (63.6 ± 28.5%) above 50%, whereas a group that received separately pulsed BMDC (Gr IV) completely failed to develop blocking activity in the serum (mean blocking titer 1.8 ± 0.8%) (Figure 8(a)). A group immunized with GII.4-pulsed BMDCs (Gr II) resulted in mean blocking titer of 26.5 ± 11.8% with only one mouse having a blocking index over 50%. The assay was repeated using groupwise pooled sera, which confirmed that only a group that received double-pulsed BMDCs (Gr III) developed a considerable blocking activity in the serum (Figure 8(b)). The control group (Gr I) was negative for blocking antibodies.

## 4. Discussion

The mechanisms leading to protective NoV immunity are not well characterized. However, humoral immunity seems to be especially important as blocking antibodies, memory B cells, and salivary IgA have all been shown to correlate with protection [16, 18, 49]. In order to generate a strong adaptive B cell immunity, help from CD4+ T cells is essential, and the APCs capable of binding, internalizing, and processing microbes are the key players in priming T cells [26]. We have previously studied the maturation and activation of immortalized cell lines serving as APCs in response to our NoV and RV combination vaccine antigens, GII.4 VLPs and RV VP6 [23]. However, as immortalized APCs are genetically modified, and do not represent the natural cell populations, primary BMDCs were utilized in the present study. BMDCs are an excellent model to study the immunological processes *in vitro*, as they are relatively easy to obtain and sustain, and can be pulsed *in vitro* to be transferred back to syngeneic mice [29, 44, 50, 51]. However, BMDCs are heterogenic cell population, comprised of many cell types in addition to DCs [52]. CD11c is the best marker for DCs [44], and in our BMDC preparations, the number of CD11c+ cells was recurrently over 90%, indicating high purity. Frozen-thawed BMDCs were used in this study to reduce assay variability and the amount of labor and time, as if the BMDCs were generated from precursor cells for each assay. It has been shown that freeze thawing does not affect the uptake, processing, or antigen presentation capacity of murine BMDCs, and frozen cells retain the ability to induce antigen-specific immune responses *in vivo* [48]. We investigated the antigen uptake and functionality of GII.4 VLP- and VP6-pulsed BMDCs *in vitro*, followed by adoptive transfer of these cells to mice to study the *in vivo* immunogenicity, and in particular, the adjuvant effect of VP6.

The intracellular staining showed that both GII.4 VLPs and VP6 were uptaken by the BMDCs, which is concurrent with the previous results obtained by us and others with cell line-derived APCs [23, 53]. VP6 was internalized more efficiently than the VLPs, most likely because of the size and/or shape difference between tubular VP6 (0.2-1.5 *μ*m) and spherical GII.4 VLPs (~38 nm). Larger complexes have been shown to be preferentially uptaken by the APCs [53, 54], but also surface charge is known to affect the attractiveness of a particle to be phagocytosed [55]. Upon uptake of foreign particles, immature DCs are known to go through series of biological and phenotypical changes (such as upregulation of costimulatory and MHC II molecules) leading to maturation [26]. BMDCs are known also to be easily activated *in vitro* without microbial stimuli, e.g., in response to conditions in the propagation culture or to certain treatments [56]. Unfortunately, we could not measure the upregulation of maturation markers in response to GII.4 VLP and VP6 uptake, as we detected high baseline expression of CD80, DC86, and MHC II molecules in our untreated BMDC culture indicating spontaneous maturation. We ruled out the possible bacterial contamination of the culture as background cytokine levels in unstimulated BMDC cultures were low, but in response to LPS, robust levels of TNF-*α* and IL-6 were induced. Despite spontaneous maturation, BMDCs are known to continue capture, process, and present exogenous antigens [31, 57, 58]. Platt et al. [31] have shown that the postmaturation uptake occurs, with comparable efficacy to immature DCs, through receptor-mediated endocytosis, which is the route that we have previously shown to be (partially) responsible for VP6 uptake [23].

We also examined the functionality of the pulsed BMDCs, particularly, whether they are able to present the antigen and induce antigen-specific T and B cell responses *in vitro*. Robust IFN-*γ* secretion was detected in the ELISpot assay by antigen-primed splenocytes stimulated with GII.4 VLP- or VP6-pulsed BMDCs. IFN-*γ* production against native protein constructs was considerably lower, indicating that the pulsed BMDCs were responsible for presenting the captured and processed antigens to T cells. Given that only a small fraction of the total amount of pulsed BMDCs used in the ELISpot had internalized GII.4 VLPs (~1.6%) and VP6 (~10%), the ability of these BMDCs to stimulate T cells seems to be very powerful. Furthermore, GII.4 VLP-pulsed BMDCs triggered *in vivo*-primed splenocytes for GII.4-specific IgG production after seven days, indicating the activation of memory B cells [59]. The B cell stimulation could have occurred through CD4+ T cell activation, which subsequently drove cognate B cells into IgG production, but in addition to this, DCs can also interact directly with B cells providing signal through CD40 ligation [59, 60]. Furthermore, extrafollicular DCs and macrophages are able to uptake and retain intact antigens on cell surface or in intracellular vesicles and transfer them to naïve B cells to initiate antibody production [61]. However, at least in *in vitro* settings, GII.4 VLP-pulsed BMDCs could not directly activate naïve B cells for antibody production, as the coculture with naïve mouse splenocytes was negative for GII.4-specific antibody.

The repetitive surface structure of VLPs efficiently cross-links B cell receptors leading to strong humoral responses [62] but also facilitates the phagocytosis of these particles by professional APCs [63]. However, studies with certain VLPs have shown that although VLPs are readily and efficiently internalized by the DCs, they fail to trigger DC activation [30, 32]. The lack of activation might be a result of the absence of additional stimuli (such as viral RNA) to cells of innate immunity [64, 65], suggesting that some VLPs might require an external stimulator to efficiently initiate DC-driven immune responses. In an earlier study, we observed that coadministered RV VP6 promoted GII.4 VLP internalization and activated macrophages more efficiently than VLPs alone [23]. We reasoned that VP6 enhances VLP-specific responses through depo effect acting as a delivery vehicle and also by stimulating inflammatory cytokine production [23]. Here, we demonstrated that GII.4 VLP and VP6 enter in the same BMDCs when mixed in the same pulsing reaction. The uptake of GII.4 VLPs was enhanced by 37% when codelivered with VP6, which is almost exactly the same result as obtained with RAW macrophages (30% enhancement, respectively), although the number of VLP uptaken BMDCs is much lower compared to RAW cells [23]. Also, concurrent with the previous study [23], we noticed enhanced TNF-*α* and IL-6 production in cultures after pulsing GII.4 VLPs with VP6 in comparison to solely VLP-pulsed BMDCs. TNF-*α* is an important inflammatory cytokine, which recruits other APCs to inflammation site and facilitates their trafficking in draining lymph nodes [66]. In addition, TNF-*α* and IL-6 provide B cells proliferation and activation stimuli [67]. The results obtained here and previously, with immortalized cell lines as APCs, suggest that coadministration of VP6 might serve the additional stimulus needed to more efficiently activate APCs with VLP-based vaccine.

After confirming the APC functionality of GII.4 VLP- and RV VP6-pulsed BMDCs *in vitro*, we investigated their function *in vivo*. We have previously shown that VP6 has to be administered at the same time and at the same site as NoV VLPs, in order to exert adjuvant effect on NoV immune responses [24]. Here, we were especially interested to examine if VP6 nanotubes have to be uptaken by the same BMDCs as GII.4 VLPs to enhance NoV-specific immune responses. In order to study that we transferred simultaneously and separately VLP- and VP6-pulsed BMDCs to mice and compared the responses to the ones induced by BMDCs pulsed only with VLPs, all study groups immunized with pulsed BMDCs developed GII.4-specific IgG antibodies, but on average, the level of IgG trended the highest in the group receiving simultaneously VLP- and VP6-pulsed BMDCs. Higher number of mice in the experimental groups might have improved the statistical significance of the results. The importance of VP6 being mixed with VLPs is supported by the fact that only the group receiving copulsed BMDCs generated antibodies with considerable blocking potential (>50% blocking index). Furthermore, a group receiving only VLP-pulsed BMDCs or separately pulsed BMDC failed to induce >50% blocking activity, indicating that the generation of blocking activity was related to double pulsing the BMDC. Whether it was due to higher levels of IgG or improved quality of antibodies in this group remains to be unraveled but supports the adjuvant effect of VP6.

IgG subtype measurement revealed that GII.4 VLP-pulsed BMDCs induced only IgG1, indicative of Th2-type response, whereas coadministration with VP6-pulsed cells resulted in more balanced IgG1/IgG2a production, indicating unbiased Th1/Th2 response. Typically, the *in vivo* immune response triggered by antigen-pulsed BMDC is shifted to Th1 type [30, 51, 68], in which T cells respond to DC contact by producing Th1-type cytokines such as IFN-*γ*. Although we showed that both VLP- and VP6-pulsed BMDCs elicited strong IFN-*γ* production in antigen-primed T cells *in vitro*, the *in vivo* conditions in initiating primary response in naïve animals differ remarkably. There could be several reasons why GII.4-pulsed BMDCs induced solely Th2-typic response. GII.4 VLPs can be uptaken by different subpopulation of BMDCs than VP6, or different pattern recognition factors (PPRs) can be involved activating distinct signaling cascades, leading to either Th1- or Th2-related cytokine production [63, 69]. As both immunization groups receiving VP6-pulsed BMDC, either simultaneously or separately pulsed with GII.4 VLPs, resulted in GII.4-specific IgG2a production, it seems likely that the uptake of VP6 modulates the cytokine environment, affecting also to VLP uptaken cells through a bystander effect. However, revealing the exact mechanism behind the skewing of the Th1/Th2 response requires additional studies.

The immune responses to pulsed BMDCs versus native protein antigens are naturally very different; as in the latter, the proteins are directly interacted with B cells and other immune cells. However, the results obtained here with *in vivo* transfer of GII.4 VLP- and VP6-pulsed BMDC further promote our earlier findings [14, 23–25] of the adjuvant effect of VP6 on NoV VLP-induced immune responses.

## 5. Conclusions

Taken together, this study showed that both NoV VLPs and RV VP6 nanotubes were internalized and processed by mouse BMDCs. Codelivered RV VP6 nanotubes enhanced the uptake of NoV VLPs and BMDC activation, which presumably further reflected in better NoV-specific humoral responses *in vivo*, when pulsed BMDCs were used to immunize syngeneic mice. The results of this study support the earlier findings of VP6 acting as a natural adjuvant for NoV-specific immune responses both *in vitro* and in *vivo*.

## Data Availability

The data used to support the findings of this study are available from the corresponding author upon a reasonable request.
